# Isolation and characterization of a human colon adenocarcinoma cell line resistant to doxorubicin.

**DOI:** 10.1038/bjc.1986.206

**Published:** 1986-09

**Authors:** M. Grandi, C. Geroni, F. C. Giuliani


Br. J. Cancer (1986), **54**, 515–518

### Short Communication

### Isolation and characterization of a human colon adenocarcinoma cell line resistant to doxorubicin

M. Grandi, C. Geroni & F.C. Giuliani

*Biological Research and Development, Farmitalia-C.Erba Research Center, Via Giovanni XXIII, 23, 20014 Nerviano, Milano, Italy.*

Resistance to doxorubicin (DX) frequently emerges in patients, limiting its use in repeated courses of treatment (Curt *et al.*, 1984; Goldie & Coldman, 1984). Several authors (for review, see Kaye & Merry, 1985) have demonstrated that one mechanism of resistance to DX involves impaired accumulation and retention of the drug, and that DX-resistant cells are also resistant to compounds having different chemical structures and modes of action.

This has been reported *in vivo* in murine tumours and *in vitro* in established cell lines from animal and human sources. In cell lines isolated from patients (Shoemaker, 1983; Merry *et al.*, 1986; Louie *et al.*, 1986) multi-drug resistance has been reported, whereas reduced drug accumulation has not been observed. Another characteristic of DX-resistance, possibly related to reduced drug accumulation, is enhanced production of a membrane glycoprotein with a molecular weight ~170,000 daltons. This has been reported in some resistant cell lines (Kartner *et al.*, 1985; Bhalla *et al.*, 1985; Roninson *et al.*, 1984). Gene amplification is at the origin of this overproduction, as already observed in cells with other resistance mechanisms (Starks & Wahl, 1983).

We selected a human colon adenocarcinoma cell line (LoVo) isolated from a metastatic nodule for studying DX-resistance; its morphology and biochemical characteristics have been fully described (Drewinko *et al.*, 1976, 1984).

A DX-resistant subline (LoVo/DX), about 25–30 times more resistant to DX than LoVo, was obtained *in vitro* after 5–6 treatments with DX. It presents the characteristics we have summarized *viz.*, reduced accumulation of DX, and cross-resistance to different antitumour substances. A protein which is overexpressed has also been observed (M. Ciomei, unpublished).

Correspondence: M. Grandi.
Received 17 February 1986; and in revised form, 3 June 1986.

We also report here the cytotoxic activity and intracellular drug accumulation of two anthracycline analogues bearing an iodine atom in position 4 of the aminosugar.

LoVo and LoVo/DX were cultured at 37°C in Ham's F12 medium supplemented with 20% foetal calf serum, 1% of a 200 mM glutamine solution, $100\,U\,ml^{-1}$ penicillin, $100\,\mu g\,ml^{-1}$ streptomycin and 1% of a BME vitamin solution 100X. Cells were passaged every 3–4 days and maintained at 37°C in an atmosphere of 5% $CO_2$.

LoVo/Dx was obtained as follows: exponentially growing cultures were treated with $100\,ng\,ml^{-1}$ DX; every 2–3 weeks the medium was substituted with fresh medium containing $100\,ng\,ml^{-1}$ DX. After 5–6 courses of treatment, cells were harvested with 0.25% trypsin and cloned in plastic wells in the presence of the same amount of drug. LoVo/DX is derived from a single-cell colony and was continuously maintained in $100\,ng\,ml^{-1}$ DX.

The experiments described were performed after 30 passages in drug-containing medium. Doubling time was determined by seeding LoVo and LoVo/DX at concentrations of $2\times10^4$ and $4\times10^4$ cells/dish in 36 mm plastic dishes (2 ml/dish). Every 24 h two replicate samples were harvested with 0.25% trypsin and resuspended in 2 ml growth medium: Cell number was determined with a Coulter Counter (Mod. ZM). Plating efficiency was measured by plating cell concentrations ranging from 100 to 1500 cells/dish; colonies with $\geq 50$ cells/colony were counted under an inverted microscope after 8–10 days.

*Clonogenic assay* A single-cell plating technique (Howell *et al.*, 1984; Miyamoto *et al.*, 1984) was employed to assess cytotoxicity. Exponentially growing cultures were harvested with 0.25% trypsin and resuspended in growth medium; 400 cells/plastic dish (36 mm) were seeded 24 h before treatment to permit the cells to adhere to the plastic surface. Cells were exposed to the drugs by replacing the growth medium with drug-containing medium; exposure was 1 or 24 h at 37°C, then medium was withdrawn, attached cells were rinsed

© The Macmillan Press Ltd., 1986

once with saline, and fresh growth medium was added. Colony number was determined after 8–10 days incubation at 37°C. The 50% inhibiting dose ($ID_{50}$) was calculated from dose-response curves. The resistance index (RI) is the ratio between $ID_{50}$ values on LoVo/DX *vs.* LoVo.

*Intracellular drug accumulation* Cells were seeded at the concentration of $1.2 \times 10^6$ cells/dish (60 mm, 4 ml) and incubated at 37°C for 24 h before treatment; for drug exposure, growth medium was withdrawn and replaced with medium containing 2.5 $\mu g\,ml^{-1}$ of DX, daunorubicin (DNR), 4′-deoxy-4′-I-doxorubicin (4′-I-DX) and 4′-deoxy-4′-I-daunorubicin (4′-I-DNR) (Figure 1). Exposure was for 15, 30, and 60 min at 37°C; then medium was withdrawn, and cells were quickly washed twice with ice cold saline and harvested with a few drops of 0.25% trypsin at room temperature. Detached cells were suspended in 3 ml ice cold saline, collected by low-speed centrifugation at 4°C and resuspended in 1 ml of a 1:1 mixture of ethanol: 0.3N HCl for extraction of the drugs. Intracellular

| COMPOUND | $R_1$ | $R_2$ |
|---|---|---|
| Doxorubicin | OH | OH |
| 4′-deoxy-4′-Iodo-Doxorubicin | OH | I |
| Daunorubicin | H | OH |
| 4′-deoxy-4′-Iodo-Daunorubicin | H | I |

**Figure 1** Structures of anthracyclines.

drug content was determined by fluorescence spectrophotometry (excitation and emission wavelengths respectively 479 and 593 nm) and is reported as $\mu g$ drug $10^{-6}$ cells.

Each sample was run in triplicate. Cell number was determined with a Coulter Counter on an aliquot of the cell suspension before centrifugation.

*Drugs* The anthracyclines evaluated, DX, 4′-I-DX, DNR, 4′-I-DNR were from Farmitalia C. Erba, Milan, Italy. Other antitumour drugs were: mitomycin C (Kyowa Hakko, Tokyo, Japan), vincristine suplhate (trade name ‘Oncovin’, Eli Lilly, Indianapolis, USA), aclacinomycin A (NSC 208734, from NCI), VP-16-213 (trade name ‘Vepesid’, Bristol Myers Pharmaceuticals, Slough, UK), actinomycin D (Sigma Chemical Co., St. Louis, USA) and arabinosylcytosine (Ara C, Sigma Chemical Co., St. Louis, USA). Compounds were dissolved in sterile water and stored at −20°C in 0.2 ml stocks at the concentration of 0.1 $mg\,ml^{-1}$. All subsequent dilutions were made in growth medium immediately before treatment.

*Characteristics of LoVo/DX* The doubling time of LoVo/DX and LoVo was 24 h with a lag in resuming exponential phase of 24 h for LoVo and 40 h for LoVo/DX. Plating efficiency was 50% (s.d. ± 12) for LoVo, and slightly less – 38% (s.d. ± 8.4) – for LoVo/DX.

*Cytotoxicity and intracellular content of anthracyclines* The cytotoxicity of DX, 4′-I-DX, DNR and 4′-I-DNR was measured on LoVo and LoVo/DX after 60 min exposure (Table I). LoVo/DX were resistant to DX and DNR (RIs 26.7 and 24.8 respectively) and much less resistant to the iododerivatives 4′-I-DX and 4′-I-DNR (RIs 4.4 and 3 respectively). These cytotoxicity values appear to correlate, at least qualitatively, with the intracellular content of the drugs. Figure 2 shows the net drug content determined on LoVo and LoVo/DX after short exposure. In both cell lines,

**Table I** Cytotoxic activity of DX, 4′-I-DX, DNR and 4′-I-DNR on LoVo and LoVo/DX cells after 60 min treatment.

| *Product* | *LoVo* $ID_{50}$ ($ng\,ml^{-1}$) | *LoVo/DX* $ID_{50}$ ($ng\,ml^{-1}$) | *RI* |
|---|---|---|---|
| Doxorubicin | 243 ± 15 | 6500 ± 707 | 26.7 |
| 4′-I-Doxorubicin | 13 ± 1.5 | 57.5 ± 10 | 4.4 |
| Daunorubicin | 125 ± 46 | 3100 ± 312 | 24.8 |
| 4′-I-Daunorubicin | 20 ± 8 | 60 ± 7 | 3 |

$ID_{50}$: mean ± s.d. of 3 replicate experiments and RI: resistance index.

**Figure 2** Intracellular drug of anthracyclines (a) LoVo, (b) LoVo/DX, (*) Doxorubicin; (▲) 4′-I-doxorubicin; (●) daunorubicin; and (■) 4′-I-daunorubicin. Each point represent s the mean ± s.d. of 3 replicate points.

DX and DNR reached lower intracellular levels than their iododerivatives: Drug content in LoVo/DX was however lower for all four compounds, more markedly for DX and DNR than for 4′-I-DX and 4′-I-DNR.

These two iododerivatives have been reported to be active both *in vitro* and *in vivo* on P388 murine leukaemia resistant to DX (Geroni *et al.*, 1983; Barbieri *et al.*, 1984). 4′-I-DX has been further evaluated and is a clinical candidate since it is much less cardiotoxic than DX and shows good antitumour activity (Arcamone, 1985; Barbieri *et al.*, unpublished).

*Cross-resistance studies* The cytotoxic activity of different antitumour drugs after 24 h exposure to graded concentrations of the compounds is shown in Table II. LoVo/DX cells were resistant to doxorubicin (RI, 30.4), VP-16 (RI, 25), vincristine (RI, 22.4) and actinomycin D (RI, 7.8). Lower resistance was observed for aclacinomycin A (RI, 2.87) and mitomycin C (RI, 3.1). No cross-resistance was observed with Ara-C; this compound in fact was more active on the resistant line than on the sensitive one. This 'collateral sensitivity' of DX-resistant cells to Ara-C has been observed in other studies (Johnson *et al.*, 1978; Bhalla *et al.*, 1985).

**Table II** Cytotoxic activity of different antitumour compounds on LoVo and LoVo/DX cells after 24 h exposure.

| *Product* | *LoVo* $ID_{50}{}^{a}$ *(ng ml⁻¹)* | *LoVo/DX* $ID_{50}{}^{a}$ *(ng ml⁻¹)* | *RI* |
|---|---|---|---|
| Doxorubicin | 17.5 ± 6 | 533 ± 57 | 30.4 |
| VP-16 | 20 ± 5 | 500 ± 52 | 25 |
| Vincristine | 50 ± 8.2 | 1120 ± 57 | 22.4 |
| Actinomycin D | 28 ± 11 | 220 ± 62 | 7.8 |
| Mitomycin C | 20 ± 7 | 62.5 ± 17.6 | 3.1 |
| Aclacinomycin A | 47.6 ± 7.3 | 137 ± 12 | 2.87 |
| Ara-C | 150 ± 45 | 45 ± 7 | 0.3 |

[a]$ID_{50}$: mean ± s.d. of three replicate experiments and RI: resistance index.

*Conclusions* As already shown on other human and animal DX-resistant cell lines, on LoVo/DX we observed multi-drug resistance and reduced drug content. The method we applied determines only the intracellular drug content and does not measure uptake and efflux of the drug separately. However, we observed faster drug efflux on LoVo/DX than on the sensitive line (data not shown). In any case, the comparison between LoVo and LoVo/DX clearly demonstrates a lower drug content in the resistant line.

A puzzling question is that the low intracellular drug content does not account for the marked differences in sensitivity observed in the resistant line. DX-resistance is probably a multifactorial phenomenon; in fact very recently DX-induced DNA damage was described as being reduced in the P388/DX line (Capranico *et al.*, 1986) even when DX levels were comparable. The impaired drug accumulation is certainly a fundamental effect, since the cross-resistance observed to unrelated antitumour drugs would be difficult to explain by other mechanisms. Besides, we have shown that DX-resistance can be partially overcome with anthracycline analogues able to enter resistant cells. Potentially this represents important therapeutic properties because this new class of anthracyclines might be active on tumours which respond poorly to DX, and could also be used in therapy following DX or DNR treatment.

The authors wish to thank Mr A. Dadda and Mr A. Marsiglio for excellent technical assistance.

### References

ARCAMONE, F. (1985). Properties of antitumor anthracyclines and new developments in their application: Cain Memorial Award Lecture. *Cancer Res.*, **45**, 5995.

BARBIERI, B., SUARATO, A., PENCO, S., GERONI, C., BELLINI, O., FUMAGALLI, A., CASAZZA, A.M. & GIULIANI, F.C. (1984). Biologic activity of 4′-halo anthracyclines. *Proc. Am. Assoc. Cancer Res.*, **25**, 305.

BHALLA, K., HINDENBURG, A., TAUB, R.N. & GRANT, S. (1985). Isolation and characterization of an anthracycline-resistant human leukemic cell line. *Cancer Res.*, **45**, 3657.

CAPRANICO, G., DASDIA, T. & ZUNINO, F. (1986). Comparison of doxorubicin-induced DNA damage in doxorubicin-sensitive and resistant P388 murine leukemia cells. *Int. J. Cancer* **37**, 227.

CURT, G.A., CLENDENINN, N.J. & CHABNER, B.A. (1984). Drug resistance in cancer. *Cancer Treatment Rep.*, **68**, 87.

DREWINKO, B., ROMSDAHL, M.M., YANG, L.Y., AHEARN, M.J. & TRUJILLO, J.M. (1976). Establishment of a human carcinoembryonic antigen-producing colon adenocarcinoma cell line. *Cancer Res.*, **36**, 467.

DREWINKO, B., YANG, L.Y., LEIBOVITZ, A., BARLOGIE, B., LUTZ, D., JANSSON, B., STRAGAND, J.J. & TRUJILLO, J.M. (1984). Cellular discriminants for a biological classification of human colon carcinoma. *Cancer Res.*, **44**, 4241.

GERONI, C., PODESTÀ, A., BARBIERI, B., GIULIANI, F.C. & CASAZZA, A.M. (1983). Evaluation of new anthracyclines on P388 leukemia sensitive (P388/S) or resistant (P388/R) to Doxorubicin (DX). *Proc. Am. Assoc. Cancer Res.*, **24**, 280.

GOLDIE, J.H. & COLDMAN, A.J. (1984). The genetic origin of drug resistance in neoplasms: Implication for systemic therapy. *Cancer Res.*, **44**, 3643.

HOWELL, N., BELLI, T.A., ZACZKIEWICS, L.T. & BELLI, J.A. (1984). High-level, unstable adriamycin resistance in a Chinese hamster mutant cell line with double minute chromosomes. *Cancer Res.*, **44**, 4023.

JOHNSON, R.K., CHITNIS, M.P., EMBREY, W.M. & GREGORY, E.B. (1978). 'In vivo' characteristics of resistance and cross-resistance of an adriamycin-resistent subline of P388 leukemia. *Cancer Treatment Rep.*, **62**, 1535.

KARTNER, N., EVERNDEN-PORELLE, D., BRADLEY, G. & LING, V. (1985). Detection of P-glycoprotein in multidrug-resistant cell lines by monoclonal antibodies. *Nature*, **316**, 820.

KAYE, S. & MERRY, S. (1985). Tumour cell resistance to anthracyclines – A review. *Cancer Chemother. Pharmacol.*, **14**, 96.

LOUIE, K.G., HAMILTON, T.C., WINKER, M.A. & others (1986). Adriamycin accumulation and metabolism in adriamycin sensitive and resistant human ovarian cancer cell lines. *Biochem. Pharmacology*, **35**, 467.

MERRY, S., FETHERSTON, C.A., KAYE, S.B., FRESHNEY, R.I. & PLUMB, J.A. (1986). Resistance of human glioma to adriamycin 'in vitro': The role of membrane transport and its circumvention with verapamil. *Br. J. Cancer*, **53**, 129.

MIYAMOTO, T., WAKABAYASHI, M. & TERASIMA, T. (1984). New type of recovery in HeLa cells exposed to bleomycin. *Br. J. Cancer*, **49**, 247.

RONINSON, I.B., ABELSON, H.T., HOUSMAN, D.E., HOWELL, N. & VARSHAVSKY, A. (1984). Amplification of specific DNA sequences correlates with multidrug resistance in Chinese hamster cells. *Nature*, **309**, 626.

SHOEMAKER, R.H., CURT, G.A. & CARNEY, D.N. (1983). Evidence for multidrug-resistant cells in human tumor cell populations. *Cancer Treatment Rep.*, **67**, 883.

STARK, G.R. & WAHL, G.M. (1983). Gene amplification. *Ann. Rev. Biochem.*, **53**, 447.


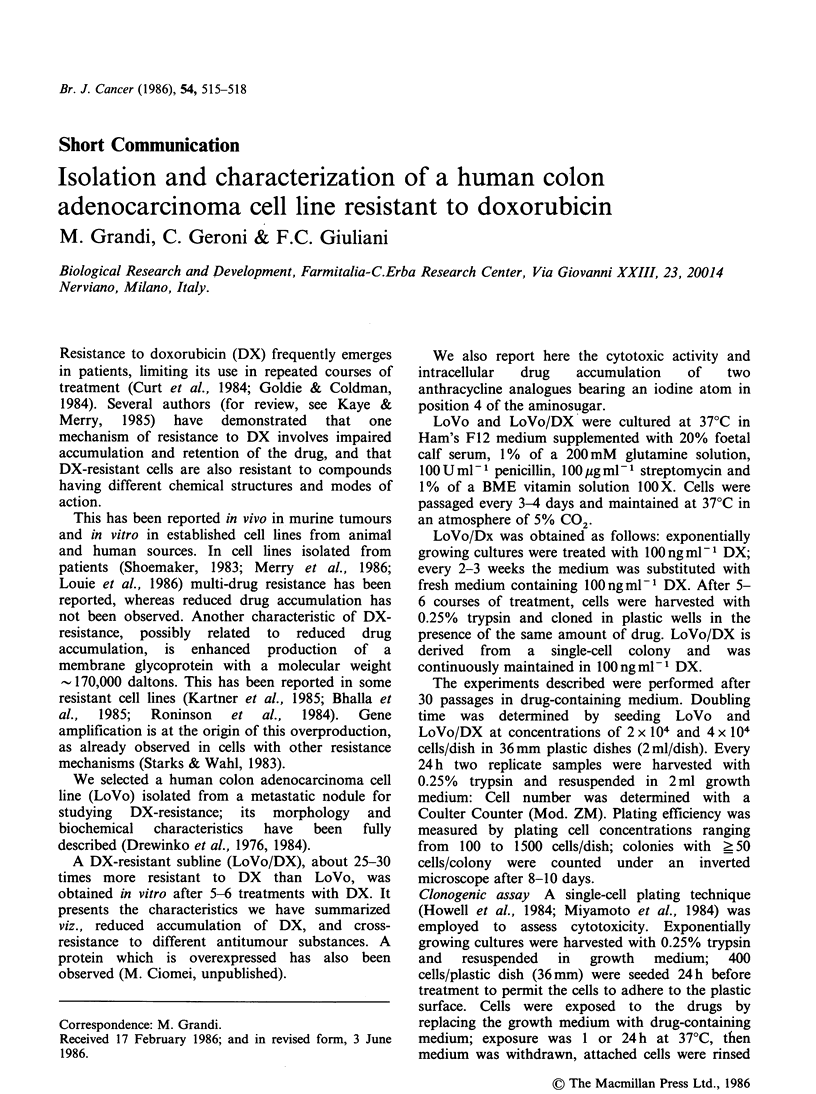

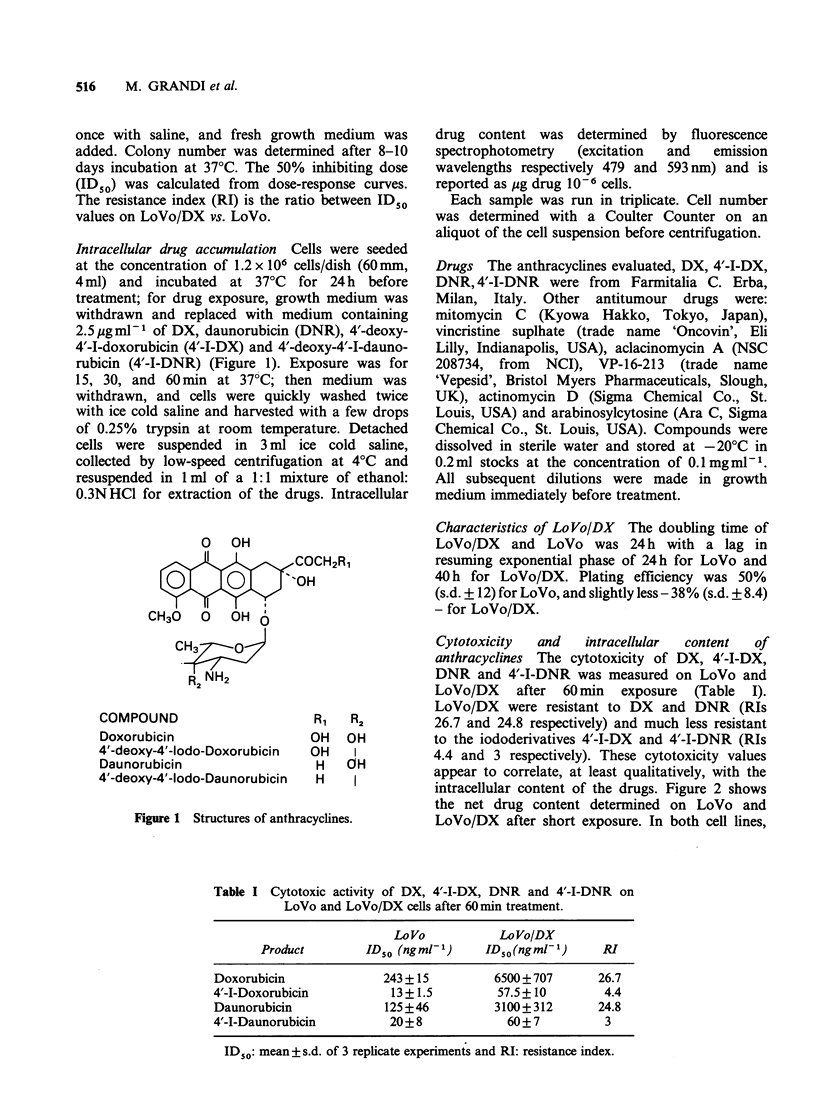

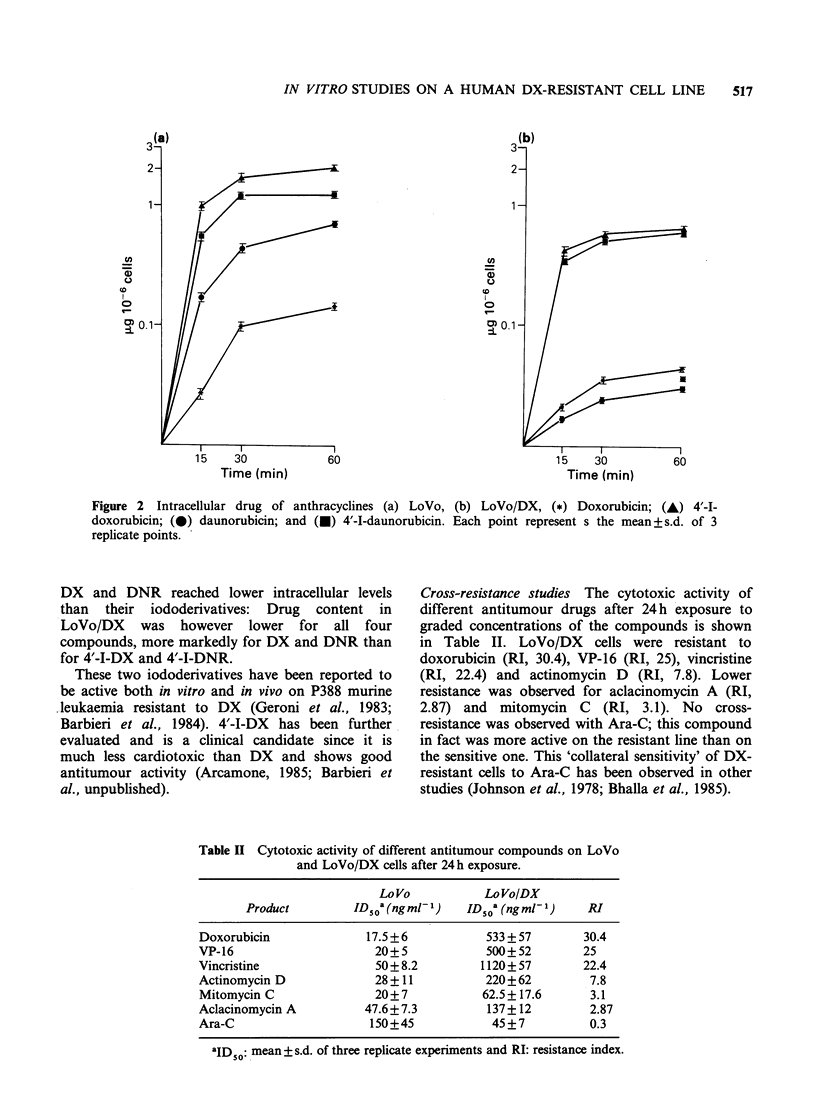

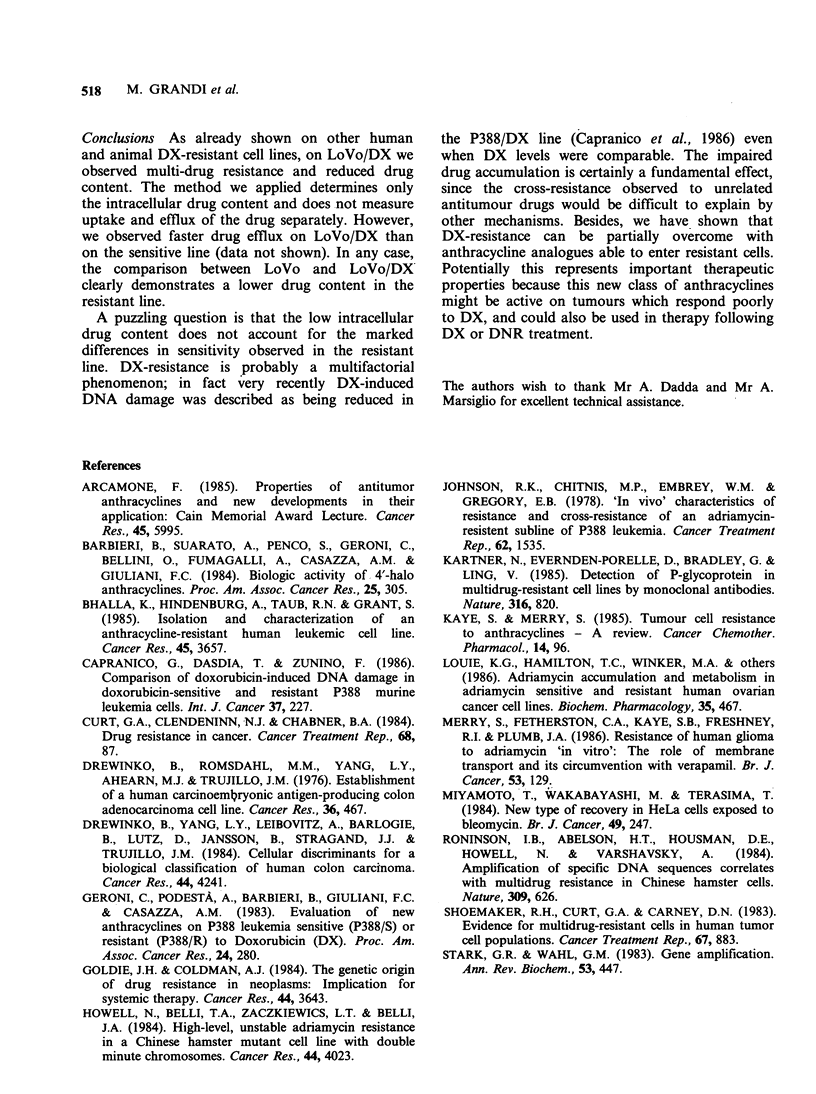


## References

[OCR_00353] Arcamone F. (1985). Properties of antitumor anthracyclines and new developments in their application: Cain memorial award lecture.. Cancer Res.

[OCR_00365] Bhalla K., Hindenburg A., Taub R. N., Grant S. (1985). Isolation and characterization of an anthracycline-resistant human leukemic cell line.. Cancer Res.

[OCR_00371] Capranico G., Dasdia T., Zunino F. (1986). Comparison of doxorubicin-induced DNA damage in doxorubicin-sensitive and -resistant P388 murine leukemia cells.. Int J Cancer.

[OCR_00377] Curt G. A., Clendeninn N. J., Chabner B. A. (1984). Drug resistance in cancer.. Cancer Treat Rep.

[OCR_00382] Drewinko B., Romsdahl M. M., Yang L. Y., Ahearn M. J., Trujillo J. M. (1976). Establishment of a human carcinoembryonic antigen-producing colon adenocarcinoma cell line.. Cancer Res.

[OCR_00388] Drewinko B., Yang L. Y., Leibovitz A., Barlogie B., Lutz D., Jansson B., Stragand J. J., Trujillo J. M. (1984). Cellular discriminants for a biological classification of human colon carcinoma.. Cancer Res.

[OCR_00402] Goldie J. H., Coldman A. J. (1984). The genetic origin of drug resistance in neoplasms: implications for systemic therapy.. Cancer Res.

[OCR_00407] Howell N., Belli T. A., Zaczkiewicz L. T., Belli J. A. (1984). High-level, unstable adriamycin resistance in a Chinese hamster mutant cell line with double minute chromosomes.. Cancer Res.

[OCR_00413] Johnson R. K., Chitnis M. P., Embrey W. M., Gregory E. B. (1978). In vivo characteristics of resistance and cross-resistance of an adriamycin-resistant subline of P388 leukemia.. Cancer Treat Rep.

[OCR_00420] Kartner N., Evernden-Porelle D., Bradley G., Ling V. Detection of P-glycoprotein in multidrug-resistant cell lines by monoclonal antibodies.. Nature.

[OCR_00426] Kaye S., Merry S. (1985). Tumour cell resistance to anthracyclines--a review.. Cancer Chemother Pharmacol.

[OCR_00431] Louie K. G., Hamilton T. C., Winker M. A., Behrens B. C., Tsuruo T., Klecker R. W., McKoy W. M., Grotzinger K. R., Myers C. E., Young R. C. (1986). Adriamycin accumulation and metabolism in adriamycin-sensitive and -resistant human ovarian cancer cell lines.. Biochem Pharmacol.

[OCR_00437] Merry S., Fetherston C. A., Kaye S. B., Freshney R. I., Plumb J. A. (1986). Resistance of human glioma to adriamycin in vitro: the role of membrane transport and its circumvention with verapamil.. Br J Cancer.

[OCR_00444] Miyamoto T., Wakabayashi M., Terasima T. (1984). New type of recovery in HeLa cells exposed to bleomycin.. Br J Cancer.

[OCR_00449] Roninson I. B., Abelson H. T., Housman D. E., Howell N., Varshavsky A. (1984). Amplification of specific DNA sequences correlates with multi-drug resistance in Chinese hamster cells.. Nature.

[OCR_00456] Shoemaker R. H., Curt G. A., Carney D. N. (1983). Evidence for multidrug-resistant cells in human tumor cell populations.. Cancer Treat Rep.

[OCR_00461] Stark G. R., Wahl G. M. (1984). Gene amplification.. Annu Rev Biochem.

